# Iatrogenic Leptomeningeal Carcinomatosis Following Craniotomy for Resection of Metastatic Serous Ovarian Carcinoma: A Systematic Literature Review and Case Report

**DOI:** 10.3389/fsurg.2022.850050

**Published:** 2022-04-25

**Authors:** Brittany M. Stopa, Joshua A. Cuoco, Srijan Adhikari, Douglas J. Grider, Cara M. Rogers, Eric A. Marvin

**Affiliations:** ^1^Virginia Tech Carilion School of Medicine, Roanoke, VA, United States; ^2^Section of Neurosurgery, Carilion Clinic, Roanoke, VA, United States; ^3^School of Neuroscience, Virginia Polytechnic Institute and State University, Blacksburg, VA, United States; ^4^Dominion Pathology Associates, Roanoke, VA, United States

**Keywords:** leptomeningeal carcinomatosis, neoplastic meningitis, ovarian cancer, craniotomy, metastases, case report, literature review

## Abstract

Metastasis of ovarian carcinoma to the central nervous system occurs in <2% of cases and classically localizes within the brain parenchyma. Moreover, leptomeningeal spread of these tumors is an exceedingly rare phenomenon. Here, we conduct a systematic review of the current literature on the natural history, treatment options, and proposed pathogenic mechanisms of leptomeningeal carcinomatosis in ovarian carcinoma. We also report a case of a 67-year-old female with stage IV metastatic ovarian serous carcinoma initially confined to the peritoneal cavity with a stable disease burden over the course of three years. Follow-up imaging demonstrated an intracranial lesion, which was resected via craniotomy, and pathology was consistent with the original diagnosis. Three months after surgery, she developed rapidly progressive dizziness, generalized weakness, fatigue, and ataxia. Repeat MRI demonstrated interval development of extensive and diffusely enhancing dural nodularity, numerous avidly enhancing supratentorial and infratentorial lesions, enhancement of the bilateral trigeminal nerves, internal auditory canals, and exit wound from the surgical site into the posterior aspect of the right-sided neck musculature consistent with diffuse leptomeningeal dissemination. The present case highlights that leptomeningeal dissemination of ovarian carcinoma is a potential yet rare consequence following surgical resection of an ovarian parenchymal metastasis. Progressive clinical symptomatology that develops postoperatively in this patient population should prompt urgent workup to rule out leptomeningeal disease and an expedited radiation oncology consultation if identified.

## Introduction

Ovarian cancer is the second most common gynecologic malignancy as well as the fifth leading cause of cancer deaths in women (1). Diagnosis typically occurs late in the disease course with patients presenting with stage III or IV disease due to a lack of effective screening methodologies and nonspecific presenting symptomatology (1). Specifically, high-grade serous ovarian carcinoma is associated with poor clinical outcomes. More than 70% of patients present with an advanced disease burden and distant metastases (2). Disease dissemination mainly involves the peritoneal cavity, with hematogenous spread rarely seen (2). Ovarian metastases to the brain are a known phenomenon, although they represent less than 2% of cases (3–8). Moreover, leptomeningeal seeding of ovarian carcinoma is an exceedingly rare entity with most cases published as case series or individual case reports (5, 9–11). As such, the literature on the natural history and effective treatment strategies of this phenomenon remains sparse.

Despite adjuvant treatment, the five-year survival for patients with ovarian carcinoma is 48%, which is attributable to disease relapse and drug resistance (2). Moreover, the five-year survival for patients with distant lesions is 29%, compared to up to a 5-year survival rate of 92% in patients with localized disease (2). Traditionally, clinicopathologic prognostic factors have not been sufficient in predicting distant spread as no molecular markers have shown to be associated with a tendency to metastasize (2, 12, 13). However, several recent studies have shown promise in identifying various biomarkers for distinguishing distant metastases in patients with high-grade serous ovarian carcinoma (2, 12, 13). Romani et al. (12) demonstrated reduced expression of claudin-7, a major component of the apical tight junction complex, to be associated with the burden of distant metastases. Moreover, Wu et al. (13) found soluble mesothelin-related peptide to be a promising biomarker of high-grade serous ovarian carcinoma with a higher specificity, omission diagnostic rate, and positive predictive value but lower sensitivity and negative predictive value compared to CA-125. Although these studies have begun to lay the groundwork for biomarker detection of distant metastases, the recognition of intracranial involvement remains a challenge due to the permeability of the blood–brain barrier. Here, we report a case of leptomeningeal carcinomatosis of ovarian serous carcinoma three months following craniotomy for resection of a metastatic intracranial lesion. We conduct a systematic review of the current literature on the natural history, treatment options, and proposed pathogenic mechanisms of leptomeningeal carcinomatosis in ovarian carcinoma.

## Case Report

**Figure 1:**
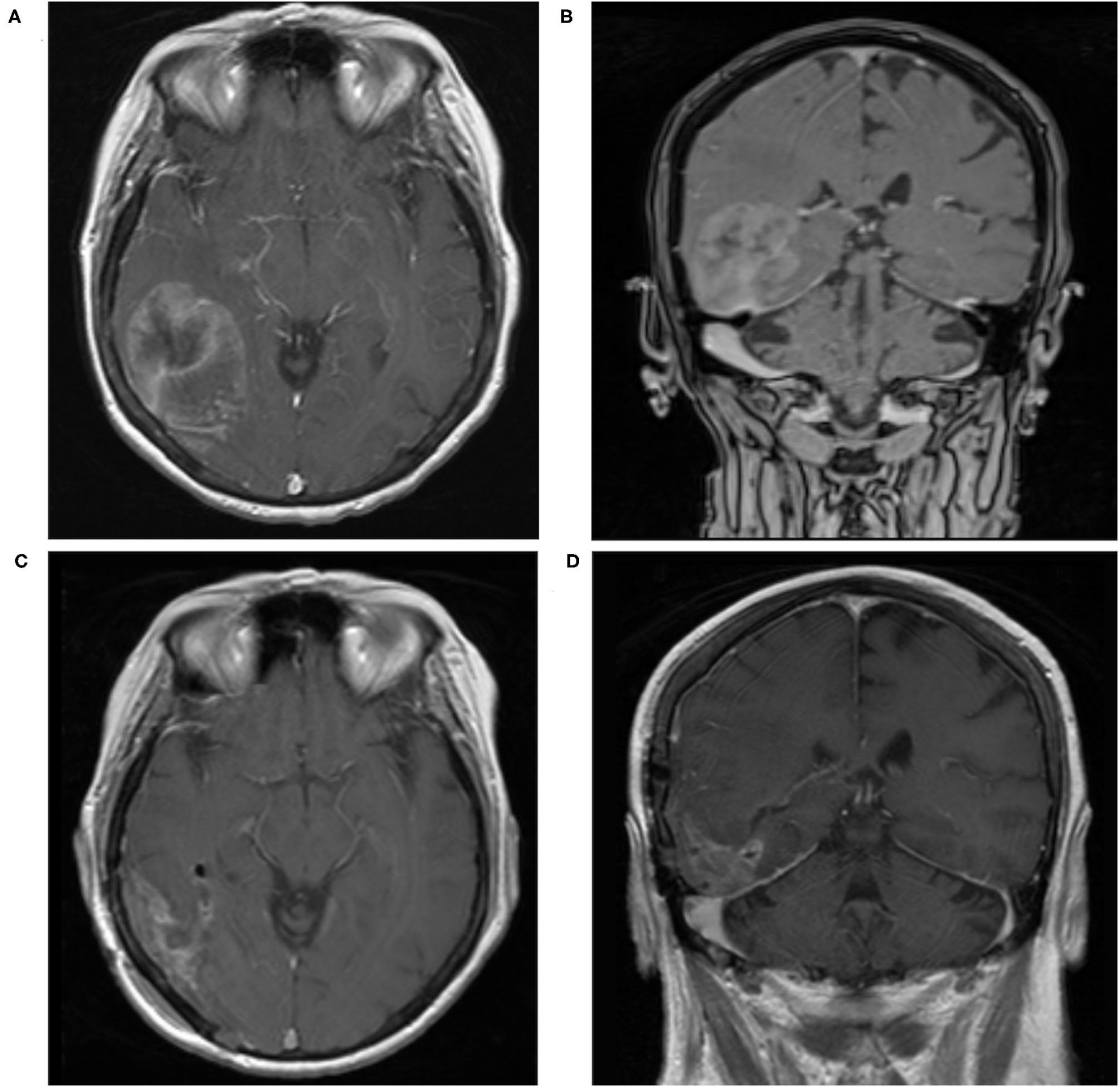
**(A,B)** Initial T1-weighted MRI with contrast demonstrates a large right posterior temporal heterogeneously enhancing lesion with scattered calcifications and surrounding vasogenic edema that appeared to arise from the right tentorium. **(C,D)** Postoperative T1-weighted MRI with contrast demonstrates expected postoperative changes with minimal residual along the tentorium.

A 67-year-old female presented to the emergency department with several days of progressively worsening headaches, dizziness, and confusion. Past medical history was remarkable for stage IV high-grade serous ovarian carcinoma diagnosed three years prior. At that time, she underwent five cycles of neoadjuvant chemotherapy followed by diagnostic laparoscopy, exploratory laparotomy, total abdominal hysterectomy, bilateral salpingo-oophorectomy, infragastric omentectomy, bilateral pelvic and para-aortic lymphadenectomy, argon bean coagulation of peritoneal implants, and optimal interval reductive surgery to no gross residual disease. She was dispositioned to postoperative chemotherapy with three cycles of paclitaxel and carboplatin. She completed the aforementioned treatment course three years prior to her presentation with no evidence of recurrent disease in the interim based on radiographic imaging and CA-125 serum levels. Given the nature of her symptomatology, there was a concern for disease recurrence with metastases to the brain prompting MRI, which demonstrated a large right posterior temporal heterogeneously enhancing lesion with scattered calcifications and surrounding vasogenic edema that appeared to arise from the right tentorium (Figures 1A,B). Repeat CT of the chest, abdomen, and pelvis was without evidence of recurrence of her primary disease or additional metastatic burden. The intracranial lesion was suspected to be metastatic in etiology rather than a new primary lesion, given her oncologic history.

**Figure 2:**
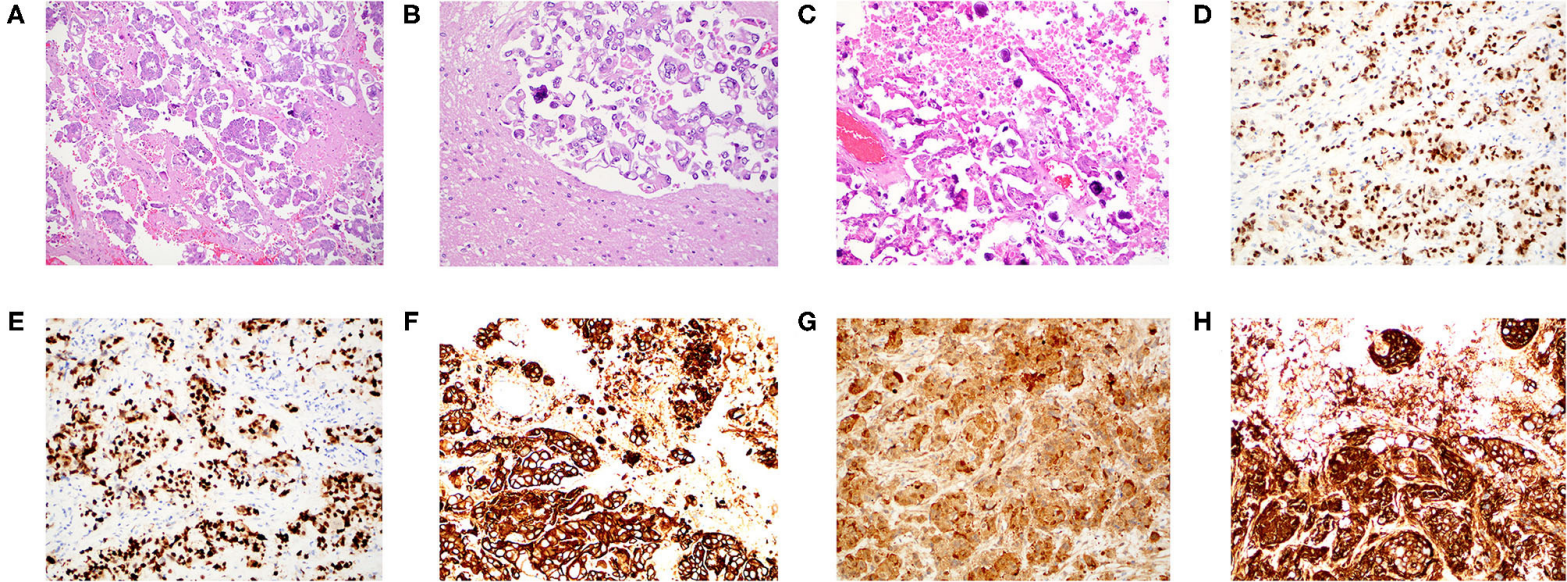
Histopathology and immunohistochemistry of the intracranial metastatic serous carcinoma. **(A)** H&E stain at 100x magnification with nests and small papillae of pleomorphic tumor cells infiltrating brain parenchyma. **(B)** H&E stain at 200 × magnification demonstrating pleomorphic carcinoma with a well-formed psammoma body. **(C)** H&E stain at 200 × magnification demonstrating numerous psammoma bodies. **(D)** WT-1 immunostain at 200 × magnification demonstrating strong nuclear positivity. **(E)** p53 immunostain at 200 × magnification strongly marking the nuclei of all tumor cells. **(F)** Cytokeratin-7 immunostain at 200 × magnification with strong cytoplasmic reactivity. **(G)** p16 immunostain at 200 × magnification with strong nuclear positivity. **(H)** CA-125 immunostain at 200 × magnification with strong cytoplasmic reactivity.

Given her clinical history and tumor size, surgical resection was recommended. A stereotactic right temporoparietal craniotomy was performed for surgical resection. The dura was opened in a C-shaped fashion toward the transverse sinus, which was skeletonized. Abnormal tissue was immediately identified along the inferior aspect of the craniotomy. The tissue was adherent to the dura and appeared to be somewhat calcified. A circumferential dissection was performed around the tumor with decent white matter planes. The tentorial pedicle was cauterized and cut sharply. There was tumor on the lateral ventricular wall, which was resected. The tumor was resected *en bloc*. There was no evidence of cerebrospinal fluid egress to suggest gross violation of the ventricular wall. Histopathology showed nests and small papillae of pleomorphic tumor cells, abundant psammoma bodies, and extensive necrosis (Figures 2A–C). Immunohistochemistry demonstrated WT-1, p53, cytokeratin-7, p16, and CA-125 positivity (Figures 2D–H). The intracranial lesion was morphologically similar to her omental biopsy three years prior with an identical immunoprofile upon comparison (Supplementary Figures 1A–H). A diagnosis of metastatic high-grade serous ovarian carcinoma based upon the intracranial lesion's histopathology, immunohistochemical staining profile, and similarity to the prior omental biopsy was made (14–18).

**Figure 3:**
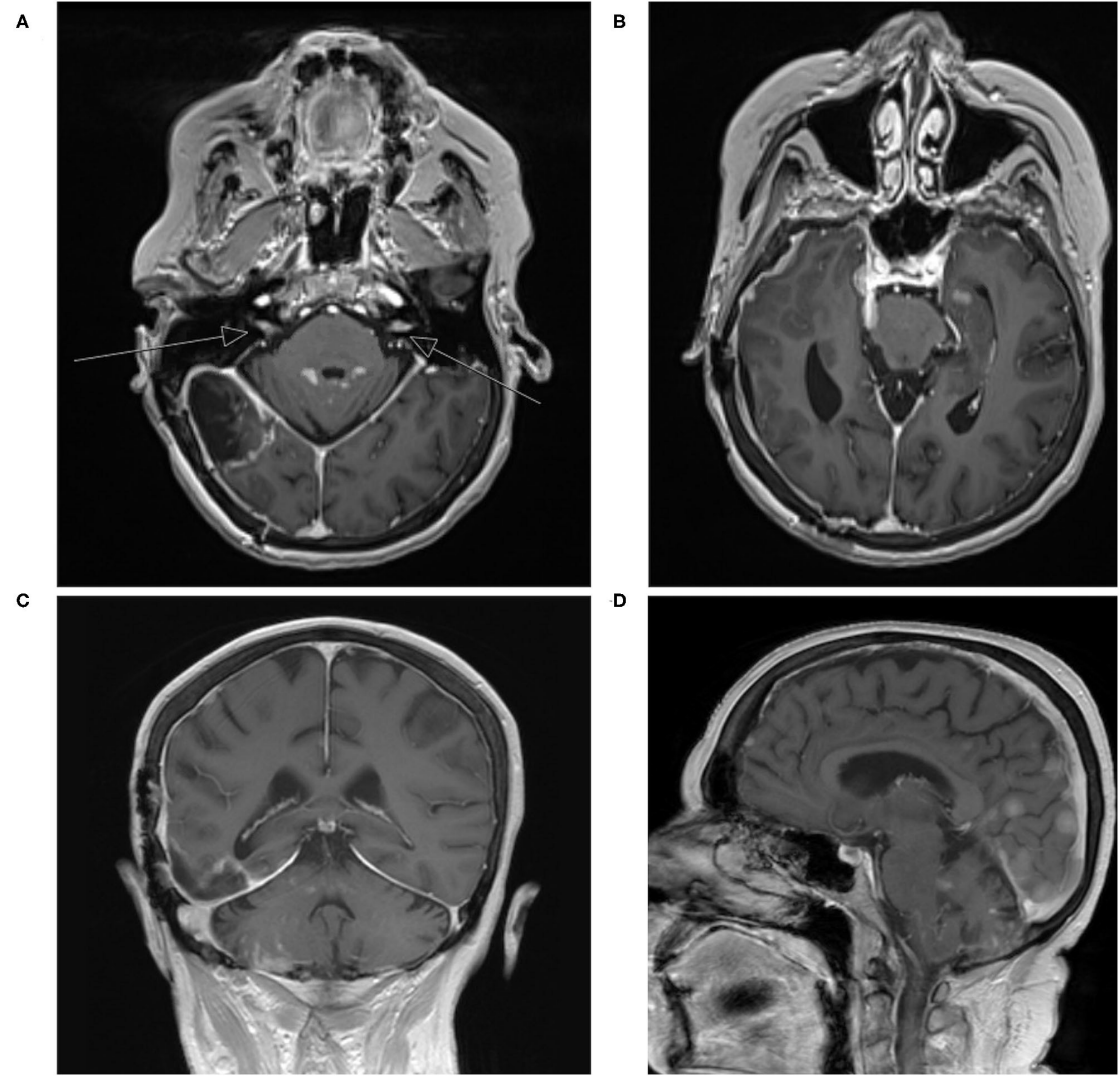
**(A–D)** Repeat T1-weighted MRI with contrast three years later with interval development of extensive diffuse enhancing dural nodularity, numerous avidly enhancing supratentorial and infratentorial lesions, enhancement of the bilateral trigeminal nerves, internal auditory canals, and exit wound from the surgical site into the posterior aspect of the right-sided neck musculature consistent with diffuse leptomeningeal dissemination (arrows indicate internal auditory canals).

Postoperative MRI of the brain demonstrated expected changes with minimal residual along the tentorium (Figures 1C,D). Her neurologic symptomatology resolved postoperatively. She completed adjuvant CyberKnife stereotactic radiation to the surgical site. Three months after surgery, she developed rapidly progressive dizziness, generalized weakness, fatigue, and ataxia. Repeat MRI demonstrated interval development of extensive diffusely enhancing dural nodularity, numerous avidly enhancing supratentorial and infratentorial lesions, enhancement of the bilateral trigeminal nerves, internal auditory canals, and exit wound from the surgical site into the posterior aspect of the right-sided neck musculature, consistent with diffuse leptomeningeal dissemination (Figure 3). This repeat MRI was obtained approximately three weeks before the patient expired. Radiation oncology provided expedited whole-brain radiation; however, she was not a candidate for intrathecal chemotherapy given her rapid disease evolution and progressive neurologic symptomatology. She was transitioned to palliative care and expired a few weeks thereafter.

## Methods

A Siemens 1.5 Tesla MRI was utilized to acquire all of the images. The following machine parameters were utilized for sequences: T1–repetition time: <800, time to echo: <30, flip angle: 90°; T2–repetition time: >2,000, time to echo: >80, flip angle: 90°. Multiplanar, multisequence imaging of the brain was obtained before and after intravenous administration of 8 ml of Gadavist.

Immunohistochemical stains were performed on paraffin tissue sections of the omental biopsy and the right intracranial metastases. The following workflow for WT-1, p53, cytokeratin-7, p16, and CA-125 immunohistochemistry was performed: primary antibody (e.g., p53 or WT-1 antibody), antigen retrieval (e.g., immunohistochemistry-tek epitope retrieval steamer set), blocking (e.g., avidin–biotin blocking kit, streptavidin–biotin blocking kit), detection (e.g., avidin–biotin complex, labeled streptavidin–biotin), chromogen substrate (e.g., 3,3′-diaminobenzidine), counterstain (e.g., Mayer's hematoxylin), and results (e.g., staining patterns).

Catalog numbers and the supplier information for each immunohistochemical stain used for the intracranial metastasis are as follows: WT-1 (Dako, IR055), p53 (Dako, GA616), cytokeratin-7 (Dako, GA619), p16 (BioSB BSB5828), and CA-125 (Dako, GA701). This p53 antibody reacts with wild-type and mutant p53 protein. Catalog numbers and the supplier information for each immunohistochemical stain used for the omental biopsy are as follows: WT-1 (Ventana, 760-4397), p53 (Cell Marque, 453M-96), cytokeratin-7 (Cell Marque, 307M-96), p16 (Ventana, 705-4793), and CA-125 (Nova Castra, NCL-L-CA-125). This p53 antibody also reacts with wild-type and mutant p53 protein.

**Figure 4:**
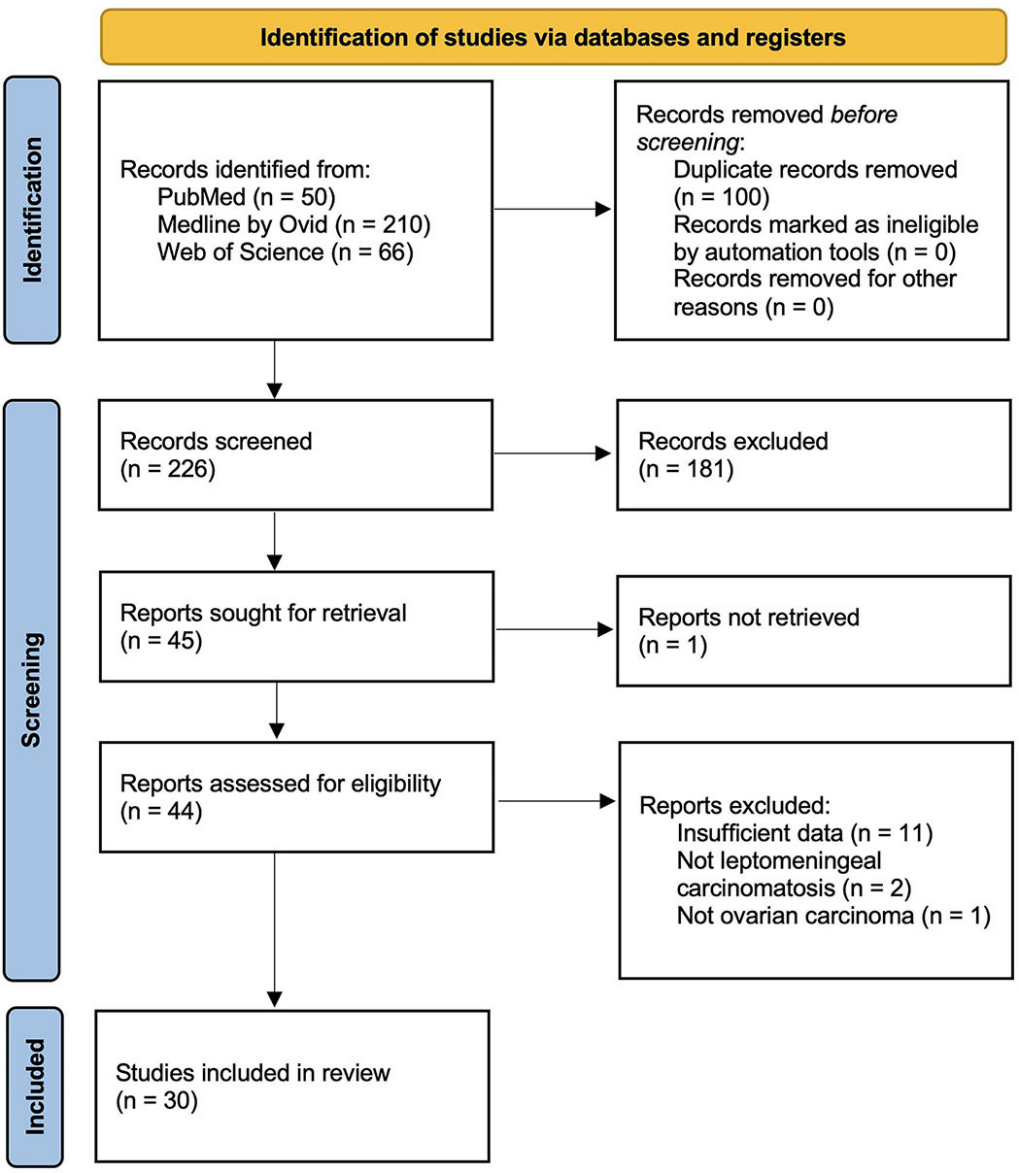
PRISMA study selection flowsheet for systematic review of the literature pertaining to leptomeningeal carcinomatosis of ovarian origin.

A systematic literature review of leptomeningeal carcinomatosis in ovarian carcinoma was performed according to PRISMA guidelines. The following databases were queried: PubMed, Ovid, and Web of Science. Search terms included “leptomeningeal” and “ovarian carcinoma.” The following terms were considered to be synonymous with leptomeningeal carcinomatosis: malignant meningitis, diffuse leptomeningeal metastasis, malignant leptomeningeal dissemination, and carcinomatous leptomeningeal metastases. Title and abstract screening and full-text screening were performed by one author (BS) with oversight and input by a second author (JC) as needed. A flowchart of the study selection is depicted in Figure 4. Eligibility criteria comprised the inclusion of case reports, case series, and cohort studies describing presentation, management, and outcomes of leptomeningeal carcinomatosis in ovarian carcinoma. Exclusion criteria included reviews and meta-analyses and reports that provided insufficient details about the clinical course and management, as well as preclinical studies. Cases were included if diagnostic evidence of leptomeningeal carcinomatosis was reported on MRI and/or cerebrospinal fluid cytology. Non-English publications were translated using Google Translate document translation service (translate.google.com) (19). Data extraction was performed by one author (BS). The collected variables included title, author, year, number of patients, primary ovarian lesion, the presence of central nervous system metastasis, time to onset of leptomeningeal carcinomatosis, presenting symptoms, treatment regimen, and survival time after diagnosis of leptomeningeal carcinomatosis. Data are qualitatively synthesized in Table 1.

**Table 1:** A systematic review of the literature pertaining to leptomeningeal carcinomatosis of ovarian origin: case report characteristics.

**References**	**Time from OC to LC (months)**	**BM before LC**	**Presenting symptoms**	**Diagnosis**		**Treatment**					**Clinical improve-** **ment**	**Expired**	**Survival from LC (months)**
				**MRI**	**CSF**	**Intra-** **thecal chemo-** **therapy**	**Oral chemo- therapy**	**Oral steroids**	**RT**	**SRS**			
al Barbarawi et al. (20)	7	1	-	1	1	0	0	0	0	0	0	1	0
Baek and Kubba (21)	108	0	Perianal focal anesthesia, sphincter dysfunction	0	1	1	1	0	0	0	0	1	8
Bangham et al. (22)	72	1	Gait imbalance, sensorineural hearing loss, trigeminal dysesthesia, perianal anesthesia, foot anesthesia	1	1	0	1	0	0	1	1	0	12
Bernstock et al. (23)	19	1	Urinary retention, gait imbalance, headache, vision changes	1	1	1	1	0	0	0	0	1	-
Chung and Allerton (24)	6	0	Nausea, vomiting, diplopia, hearing loss	-	1	0	0	0	0	0	0	1	1
Cormio et al. (25)	19	1	Gait imbalance, dizziness, radicular pain	-	1	1	1	0	0	0	1	1	15
Decelle et al. (26)	12	0	Mood changes, headache, diplopia, lethargy, nausea, ataxia	1	1	0	0	1	0	0	0	1	1
Delord et al. (27)	114	0	Paresthesia, deafness, blurred vision, dizziness, gait imbalance	0	1	1	0	0	0	0	0	1	0
Eralp et al. (28)	31	0	Headaches, nausea, vomiting, blurred vision, diplopia, gait imbalance, confusion	1	1	0	1	0	0	0	1	0	24
Favier et al. (29)	54	0	Headaches	1	1	0	1	0	1	0	1	1	20
Gleyze et al. (30)	-	0	Meningitis symptoms, upper extremity deficit	1	1	0	1	1	0	0	0	1	1
Gordon et al. (31)	14	0	Dizziness, headache, stiff neck	-	1	1	1	1	1	0	1	0	6
Goto et al. (32)	17	0	Dizziness, nausea, headache, gait imbalance	1	1	1	0	0	0	0	1	1	18
Hakim and Kamangar (33)	-	1	Altered mental status, headache	1	1	0	0	0	1	0	1	1	-
Kahn et al. (34)	2	1	Facial numbness, upper extremity paresthesia	1	1	0	1	0	0	0	0	1	3
Kawagoe et al. (35)	43	0	Dizziness, back pain, headaches	1	1	0	0	1	0	0	0	1	1
Khalil et al. (36)	25	0	Radicular pain, headache, vertigo, vomiting	-	1	1	0	1	1	0	1	1	15
Krupa and Byun (5)	-	1	Sensorineural hearing loss, facial paralysis, gait instability, low back pain with radiation	1	1	0	1	0	0	0	-	0	-
Li et al. (37)	36	0	Headaches, vomiting	1	0	0	0	0	1	0	0	1	-
Melichar et al. (38)	45	1	Seizures, headaches, gait instability, cognitive impairment	1	1	1	1	1	0	0	0	1	1
Miller et al. (11)	5	0	Blurred vision, dizziness, gait imbalance, seizure	1	1	1	0	0	1	0	0	1	4
Mukhopadhyay et al. (39)	20	0	Headaches, nausea, vomiting	1	1	1	0	0	0	0	0	1	1
Patel et al. (40)	9	0	Facial numbness, drooping eyelid, drooping mouth	0	1	1	0	0	1	0	0	1	4
Sereno Moyano et al. (41)	24	0	Memory loss, apraxia, disorientation, headache, nausea, vomiting	1	1	0	0	0	1	0	0	1	1
Stein et al. (42)	35	1	Headaches, speech difficulties, seizures	-	1	0	0	0	0	0	0	1	0
Tahir et al. (43)	9	0	Headaches, neck pain, nausea, vomiting, blurred vision, lightheadedness, syncope	0	1	1	0	0	0	0	0	1	0
Toyoshima et al. (44)	29	1	-	1	-	-	-	-	1	-	-	1	6
Toyoshima et al. (44)	42	1	-	1	-	-	-	-	0	-	-	1	1
Toyoshima et al. (44)	55	1	-	1	-	-	-	-	1	-	-	1	8
Vitaliani et al. (45)	48	0	Hearing loss, aural fullness, tinnitus, vertigo, gait imbalance	1	1	0	0	0	0	0	0	1	1
Yamakawa et al. (46)	12	0	Syncope, headache	0	1	1	0	1	0	0	1	0	7
Yildiz (47)	24	1	Headache, nausea, vomiting, altered consciousness, seizure	1	-	0	0	0	0	0	0	1	0

*BM, brain or spine metastasis; LC, leptomeningeal carcinomatosis; OC, ovarian cancer; RT, radiation therapy; SRS, stereotactic radiosurgery; 0, no; 1 yes*.

## Results of Systematic Review

A systematic review of the literature revealed 30 publications that met criteria for cases of leptomeningeal carcinomatosis in ovarian carcinoma, and a total of 32 patients were included (5, 11, 20–47). The median time to leptomeningeal carcinomatosis from ovarian carcinoma diagnosis was 24 months, and 41% of patients had brain or spine metastases present before or at the time of leptomeningeal carcinomatosis diagnosis. Presenting symptoms varied, but often included headache (17/28, 61%), gait imbalance (10/28, 36%), dizziness (6/28, 21%), and seizure (4/28, 14%) (Table 1). Leptomeningeal carcinomatosis was diagnosed on MRI in 81% (22/27) and based upon cerebrospinal fluid cytology in 96% (27/28) of cases. Treatment included intrathecal chemotherapy in 45% (13/29), oral chemotherapy in 38% (11/29), oral steroids in 24% (7/29), radiation therapy in 31% (10/32), and stereotactic radiosurgery in 3% (1/29). Overall, 32% of patients experienced some degree of clinical improvement, but ultimately 84% expired at the time of publication. The median survival time after the diagnosis of leptomeningeal carcinomatosis was one month.

## Discussion

Leptomeningeal carcinomatosis is defined as the invasion of cancer cells into the cerebrospinal fluid with subsequent seeding of the leptomeninges. Leptomeningeal carcinomatosis, also known as neoplastic meningitis, has been reported in 5–15% of patients with lymphoma and leukemia, 1–5% of patients with solid tumors, and 1–2% of patients with primary intracranial tumors (9–11). Solid tumors with the highest incidence of leptomeningeal dissemination include breast, lung, and melanoma (11). Neurologic symptomatology often includes signs of increased intracranial pressure, which may include headache, nausea, vomiting, or ambulation difficulties. Contrary to infectious meningitis, nuchal rigidity is reported in only 15% of cases with neoplastic meningitis (11).

The most common site of spread of ovarian carcinoma is the abdominal or pelvic cavities; however, metastases may involve the lymph nodes, liver, and lungs (4, 7). Other less common areas of metastatic spread include skin, spleen, thyroid, and bones (4, 7). It is exceedingly rare for primary ovarian malignancy to involve the central nervous system. Indeed, Pectasides et al. (6) found that of 1,450 patients with primary ovarian malignancies, only 17 patients (1.17%) exhibited central nervous system metastases. Moreover, none of the patients in this cohort demonstrated leptomeningeal disease (6). Cormio et al. (3) analyzed a case series of 23 patients with central nervous system metastases and found that one patient (4.34%) exhibited leptomeningeal involvement. Furthermore, Sanderson et al. (8) retrospectively analyzed the radiographic imaging of 1,222 patients with ovarian cancer. The authors reported that 13 patients (1.1%) exhibited central nervous system metastasis and two patients (0.16%) showed leptomeningeal disease (8).

Leptomeningeal seeding of ovarian carcinoma is an exceedingly rare entity with most cases published as a case series or an individual case report (5, 9–11). As such, the literature on the natural history and effective treatment strategies of this phenomenon remains sparse. Krupa et al. (5) reported a 62-year-old female with stage IIC high-grade serous ovarian carcinoma with malignant ascites but without involvement of lymph nodes who underwent bilateral salpino-oophorectomy, omentectomy, and adjuvant chemotherapy. She presented approximately one year thereafter with rapidly progressive bilateral sensorineural hearing loss, right-sided facial paralysis, gait instability, and radicular low back pain in conjunction with a marked rise in her CA-125 level (5). Imaging demonstrated bilateral auditory canal metastases with a mass extending to the right cerebellopontine angle as well as several spinal lesions with leptomeningeal involvement (5). Behnam et al. (9) described a similar rapidly progressive case in a 55-year-old female who, despite appropriate surgical intervention and adjuvant therapies for stage III serous ovarian carcinoma, developed acute onset paraparesis attributable to diffuse cranial and spinal leptomeningeal disease found on autopsy. Similarly, Miller et al. (11) reported a 49-year-old female who developed headache, vertigo, diplopia, seizures, and ataxia five months following initial diagnosis, with imaging demonstrating diffuse cranial and spinal leptomeningeal disease. Here, we report a case of a 67-year-old female who presented with several days of progressively worsening headaches, dizziness, and confusion, three months after craniotomy for resection of an ovarian carcinoma metastasis. Comparatively, the prior cases demonstrate rapidly progressive neurologic symptomatology. Retrospectively, it seems as though the presenting symptomatology is correlated to the location of leptomeningeal spread.

The pathogenic mechanism of leptomeningeal carcinomatosis is a topic of debate. Depending on the histology of the primary tumor, cancer cells may invade the meninges via one of several different mechanisms. Numerous hypotheses, including (i) hematogenous spread to the arachnoid by way of the arterial circulation or seeding of the leptomeninges through Batson's venous plexus via a retrograde venous pathway, (ii) infiltration via perineurium or endoneurium or perivascular lymphatics into dural and arachnoidal sleeves of nerve rootlets, (iii) direct extension of intraparenchymal lesions into the ventricular system or subarachnoid space, (iv) invasion of the central nervous system via the choroid plexus or arachnoidal veins, and (v) seeding of the cerebrospinal fluid from surgical removal of intraparenchymal tumors (9–11, 31, 48–50), have been proposed. Indeed, neurosurgical resection of intraparenchymal lesions permits a route of tumor spread into the cerebrospinal fluid through an iatrogenic pial breach (49). Once tumor cells gain access to the cerebrospinal fluid, they may disperse along the meningeal surface and randomly implant at distant sites forming secondary leptomeningeal metastatic lesions (49). Plaque-like deposits with accumulation in Virchow-Robin spaces and meningeal nodularity are characteristic of solid tumors whereas diffuse leptomeningeal layering is more frequently seen in hematological malignancies (49). Common areas of seeding are typically gravity-dependent anatomic areas with slow cerebrospinal fluid flow, including the posterior fossa, basilar cisterns, and cauda equina (9–11, 31, 48–50).

Although neurosurgical resection of brain metastases has become a cornerstone of oncologic treatment, studies have suggested that resection is associated with a higher risk for developing leptomeningeal dissemination (51–53). In a systematic review and meta-analysis, Tewarie et al. (54) found breast cancer origin and multiple brain metastases increased the risk of leptomeningeal spread following neurosurgical resection. Ahn et al. (55) found that metastatic lesions in proximity to the cerebrospinal fluid pathway, piecemeal resection, and the use of a Cavitron Ultrasonic Surgical Aspirator (CUSA) conferred an increased incidence of leptomeningeal carcinomatosis. Ha et al. (56) also demonstrated an association between the presence of a metastatic deposit adjacent to cerebrospinal fluid and increased risk of leptomeningeal carcinomatosis in breast cancer. Indeed, these data highlight that *en bloc* resection and avoiding violation of the ventricular wall during surgical resection are critical to circumvent an increased risk of developing leptomeningeal carcinomatosis postoperatively.

Leptomeningeal carcinomatosis has a high morbidity and mortality rate regardless of intervention. The reported median survival of leptomeningeal disease irrespective of primary diagnosis is 60 days from detection (11). Indeed, given the paucity of reported cases of leptomeningeal disease from ovarian carcinoma, there are a few clinical studies to guide treatment options. Gordon et al. (31) reported a patient with leptomeningeal spread from ovarian carcinoma who, at the time of report, survived six months after the detection with whole-brain radiation and intrathecal methotrexate. Ohta et al. (50) reported another case who survived at least 13 months after detection with whole-brain radiation, intrathecal methotrexate, and adjuvant systemic chemotherapy, including cisplatin, paclitaxel, and etoposide. Groves et al. (48) conducted a phase II trial evaluating the therapeutic efficacy of intrathecal topotecan in 62 patients with leptomeningeal carcinomatosis from a variety of primary tumors, including brain, breast, and lungs. However, only one case of ovarian cancer was included in the cohort (48). The authors found clinical improvement in 16% of patients and clinical stability in 29% of patients after a six-week induction period with intrathecal topotecan (0.4 mg two times weekly for six weeks and continued weekly for six doses) (48). Around 65% of the original cohort completed the induction period, of which 21% exhibited cerebrospinal fluid clearance of cancer cells (48). Nevertheless, the median time to progression was only seven weeks, with a median overall survival of 15 weeks (48). Miller et al. (11) utilized this protocol of intrathecal topotecan along with whole-brain radiation to treat their patient with leptomeningeal carcinomatosis of ovarian carcinoma; however, the patient continued to develop progressive symptomatology and expired four months after detection.

## Conclusions

In this report, we described a rare case of leptomeningeal carcinomatosis of metastatic ovarian serous carcinoma. The primary disease was stable for three years prior to the identification of a single metastasis to the brain parenchyma. Interestingly, our patient developed progressive neurologic symptomatology three months after craniotomy for resection of the parenchymal lesion and was subsequently found to have leptomeningeal seeding on repeat radiographic imaging. We propose that the inception of her leptomeningeal disease was iatrogenic in nature, and attributable to recent craniotomy three months prior, given the timing of her symptomatology in relation to her recent surgery. However, we do not have direct evidence to prove that leptomeningeal carcinomatosis originated from tumor spillage into the cerebrospinal fluid during neurosurgical resection rather than from systemic metastasis preoperatively. Indeed, microscopic leptomeningeal seeding imperceptible on the initial MRI cannot be definitively ruled out without preoperative cerebrospinal fluid analysis. Nevertheless, the present case highlights that leptomeningeal disease dissemination may be a rare yet potential sequela of craniotomy for the resection of intraparenchymal ovarian serous carcinoma metastases. Progressive clinical symptomatology that develops postoperatively in this patient population should prompt urgent workup to rule out leptomeningeal disease and expedited radiation oncology consultation if identified.

## Data Availability Statement

The original contributions presented in the study are included in the article/Supplementary Material, further inquiries can be directed to the corresponding author.

## Author Contributions

JC and EM conceived the study idea. SA and CR provided guidance and an oversight. BS and JC made substantial contributions to study planning and data collection, and drafted the manuscript. DG provided pathology images. All authors critically reviewed and revised this manuscript for important intellectual content.

## Conflict of Interest

The authors declare that the research was conducted in the absence of any commercial or financial relationships that could be construed as a potential conflict of interest.

## Publisher's Note

All claims expressed in this article are solely those of the authors and do not necessarily represent those of their affiliated organizations, or those of the publisher, the editors and the reviewers. Any product that may be evaluated in this article, or claim that may be made by its manufacturer, is not guaranteed or endorsed by the publisher.

## Supplementary Material

Supplementary Figure 1 Histopathology and immunohistochemistry of the original omental biopsy confirming a diagnosis of high-grade serous ovarian carcinoma. **(A)** H&E stain at 100x magnification showing high-grade carcinoma in a desmoplastic stroma. **(B)** H&E stain at 200 × magnification showing a closer view of the malignant carcinoma in nests with a hint of papillae formation in a desmoplastic stroma. **(C)** H&E stain of the surface of the ovary at 100x magnification demonstrating numerous psammoma bodies. **(D)** WT-1 immunostain at 200 × magnification demonstrating strong nuclear positivity in tumor cells. **(E)** p53 immunostain at 200 × magnification strongly marking all the tumor cell nuclei. **(F)** Cytokeratin-7 immunostain at 200 × magnification with strong cytoplasmic positivity. **(G)** p16 immunostain at 200 × magnification with strong nuclear positivity. **(H)** CA-125 immunostain at 200 × magnification with strong cytoplasmic positivity.
