# Supplementary figures and images for: Iatrogenic Leptomeningeal Carcinomatosis Following Craniotomy for Resection of Metastatic Serous Ovarian Carcinoma: A Systematic Literature Review and Case Report
Source: Front Surg. 2022 Apr 25;9:850050. doi: 10.3389/fsurg.2022.850050 (PMC9082594; doi:10.3389/fsurg.2022.850050)

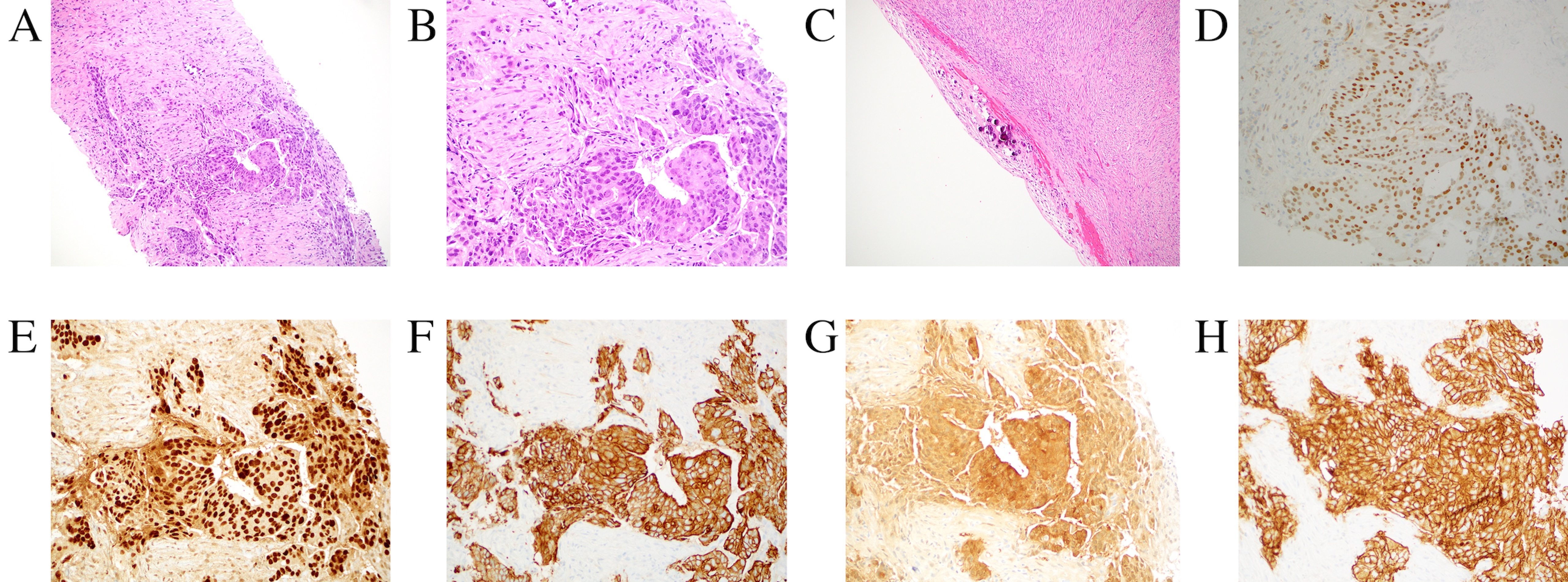

Supplement: Supplementary Figure 1 — Histopathology and immunohistochemistry of the original omental biopsy confirming a diagnosis of high-grade serous ovarian carcinoma. (A) H&E stain at 100x magnification showing high-grade carcinoma in a desmoplastic stroma. (B) H&E stain at 200 × magnification showing a closer view of the malignant carcinoma in nests with a hint of papillae formation in a desmoplastic stroma. (C) H&E stain of the surface of the ovary at 100x magnification demonstrating numerous psammoma bodies. (D) WT-1 immunostain at 200 × magnification demonstrating strong nuclear positivity in tumor cells. (E) p53 immunostain at 200 × magnification strongly marking all the tumor cell nuclei. (F) Cytokeratin-7 immunostain at 200 × magnification with strong cytoplasmic positivity. (G) p16 immunostain at 200 × magnification with strong nuclear positivity. (H) CA-125 immunostain at 200 × magnification with strong cytoplasmic positivity. [file Image_1.JPEG]
